# Binding affinity improvement analysis of multiple-mutant Omicron on 2019-nCov to human ACE2 by in silico predictions

**DOI:** 10.1007/s00894-023-05536-1

**Published:** 2023-04-24

**Authors:** Bo Li, Jindan Guo, Wenxiang Hu, Yubao Chen

**Affiliations:** 1 School of Chemistry and Environmental Engineering, Wuhan Institute of Technology, Wuhan, 430205 China; 2 NHC Key Laboratory of Human Disease Comparative Medicine, Beijing Engineering Research Center for Experimental Animal Models of Human Critical Diseases, Institute of Laboratory Animal Sciences, Chinese Academy of Medical Sciences & Peking Union Medical College, Beijing, 100021 China

**Keywords:** ACE2 receptor, Binding affinity

## Abstract

**Context:**

Since the outbreak of COVID-19 in 2019, the 2019-nCov coronavirus has appeared diverse mutational characteristics due to its own flexible conformation. One multiple-mutant strain (Omicron) with surprisingly infective activity outburst, and affected the biological activities of current drugs and vaccines, making the epidemic significantly difficult to prevent and control, and seriously threaten health around the world. Importunately exploration of mutant characteristics for novel coronavirus Omicron can supply strong theoretical guidance for learning binding mechanism of mutant viruses. What’s more, full acknowledgement of key mutated-residues on Omicron strain can provide new methodology of the novel pathogenic mechanism to human ACE2 receptor, as well as the subsequent vaccine development.

**Methods:**

In this research, 3D structures of 32 single-point mutations of 2019-nCov were firstly constructed, and 32-sites multiple-mutant Omicron were finally obtained based one the wild-type virus by homology modeling method. One total number of 33 2019-nCov/ACE2 complex systems were acquired by protein-protein docking, and optimized by using preliminary molecular dynamic simulations. Binding free energies between each 2019-nCov mutation system and human ACE2 receptor were calculated, and corresponding binding patterns especially the regions adjacent to mutation site were analyzed. The results indicated that one total number of 6 mutated sites on the Omicron strain played crucial role in improving binding capacities from 2019-nCov to ACE2 protein. Subsequently, we performed long-term molecular dynamic simulations and protein-protein binding energy analysis for the selected 6 mutations. 3 infected individuals, the mutants T478K, Q493R and G496S with lower binding energies -66.36, -67.98 and -67.09 kcal/mol also presents the high infectivity. These findings indicated that the 3 mutations T478K, Q493R and G496S play the crucial roles in enhancing binding affinity of Omicron to human ACE2 protein. All these results illuminate important theoretical guidance for future virus detection of the Omicron epidemic, drug research and vaccine development.

## Introduction

Since the breakout of coronavirus disease 2019 (COVID-19) reported on December 8, 2019, millions of people have been infected and more than 3.3 million people were killed, making this epidemic difficult to prevent and dangerous to the human beings [1]. Currently, numerous studies have been conducted on COVID-19 [2–4], and there have been significant scientific achievements including effective drugs (molnupiravir and paxlovid) [5, 6] and vaccines [7–9] which are authorized for use during emergency needs.

It is worth noting that the virus has shown multi-point mutational characteristics and can significantly affect the coronavirus activity function and even affect the current drug and vaccines’ function, significantly increasing the difficulty of epidemic prevention and control, and seriously threatening the human health and life safety of the world. One new variant of the novel coronavirus B.1.1 has been detected in South Africa, triggering rapid increase positive tested cases [10]. Preliminary studies by British scientists found that compared with wild-type SARS-nCov, the local B.1.1.7 strain is 70% stronger and infective, making the virus more difficult to prevent [11]. The new Delta variant AY.4.2 is highly contagious, more than 200% as contagious as previous variants [12]. In September 2020, a new SARS-CoV-2 variant in Denmark was detected, which can reduce the neutralization and immune protection effects of vaccine and may even abolish the current novel coronavirus vaccine [13]. The first B.1.617 double-mutant (E484Q and L452R) found in India in March 2021 was more infective and able to evade neutralizing antibody recognition, reducing the effectiveness of existing vaccines [14].

What is more, one new mutant with >50 mutations (Omicron) was first reported in South Africa. Moreover, 60% of these mutations occur on the spike region that play critical roles in binding to human cells [15]. Until December 16, the Omicron strain has appeared in more than 89 countries and regions, presenting significantly faster transmission spread than any existing mutants [16]. A research conducted by the University of Hong Kong indicated that three doses of the BNT or Pfizer vaccine could not produce enough antibody levels to fight against the Omicron variant [17]. Further investigations on the Omicron variant show milder infective properties than that of the Delta variant. As of 16 December 2021, the variant has been confirmed in more than 80 countries and in all continents except Antarctica [18]. The World Health Organization estimates that by mid-December, Omicron likely was in most countries in the world, whether they had detected it or not [19]. The Omicron strain is more likely to cause respiratory infections and more infectious than previous COVID-19 variants, therefore not easy to cause more harm to human beings [19]. Various researches showed that mutations located on the region of Spike protein can induce the structural property changes, suggesting that the Omicron variant may affect the binding affinities of ACE2 to the Spike protein [20–23]. All these reports suggest that the Omicron was more harmful to human beings. Therefore, the study of novel coronavirus mutation characteristics can provide stronger theoretical guidance for the binding mechanism of Omicron, as well as the subsequent vaccine development.

Herein, 35 structures (34 single-mutants affecting the spike protein and one multiple-mutant with 34 mutations) were constructed by homology modeling method. We then investigated the interaction patterns and binding affinities between ACE2 and all 36 mutants by in silico analysis. Results indicated that 15 single-point mutations including T95I, G142D, Δ211, S371L, S373P, K417N, G446S, S477N, E484A, Q498R, N501Y, Y505H, D796Y, Q954H, and N969K possess better binding energy higher than −55.18 kcal/mol, while 6 mutants (G339D, Q493R, G496S, S375F, N440K, and T478K) only possess lower than −60.00 kcal/mol and were selected for further binding mode analysis. Subsequently, >180-ns-long molecular dynamic simulations was operated and binding energy analysis for the 6 mutations indicated that T478K, Q493R, and G496S play the crucial roles in enhancing binding affinity of Omicron to human ACE2 protein.

## Materials and methods

### Homology modeling

Multiple-mutant with 34 mutations on 2019-nCov-Spike region based on wild-type Omicron and ACE2 complex was obtained by homology modeling method. Homology models are useful to guide mutational experiments about the structure-functional relationship and reliable in predicting the conformation of the insertion or deletion. The primary structure sequence of Omicron was compared with the target protein (PDB ID: 6VXX) for sequence blasting, and select the protein most similar to the target protein as the template for homology module construction. A high sequence similarity of 95% existed between wild-type Spike and Omicron strains. Homology modeling construction was executed by using MODELLER module [24].

### Structure preparation of 184 multiple-mutants and MD simulation

Mutational process of the 3-dimensional multiple-mutant structures was performed using the PyMOL software [25]. Molecular dynamic simulations were performed to explore the dynamic and binding differences of the tertiary changes associated with the mutations. The whole protein system was parameterized with gaff and AMBER ff99SB force fields [26]. The whole protein complex was geometrically centered with a 10 Å plus cubic water box, and electrically neutralized by adding Na+ ions. The first step was heating balance process which the whole system was balanced by using the temperature control method of 100 ps. The boost balancing process was then balanced for 100 ps, and an isotropic Berendsen pressure control method was added. An unrestricted molecular dynamic simulation for free simulation phase was conducted. Temperature and pressure control methods are the same as in the previous stage. One 10-Å cut-cutoff distance between 2019-nCov and ACE2 protein was used for van der Waals and short-range electrostatic energy calculation, and the long-range electrostatic energy was calculated using the PME method. During molecular dynamic simulations, we force 1500 kcal/mol on all heavy atoms for each protein. Each positional optimization time was at least 6 ns per system.

### Binding difference calculations

Based on the 200-ns molecular dynamic simulation, at least 3000 snapshots were extracted to obtain average structure for each protein-protein system from the equilibrium trajectory, and binding mode for each mutant was analyzed. To investigate how the mutations affect the binding modes of ACE2 to the 2019-nCoV-Spike, the root mean square deviation (RMSD) [27] of heavy atoms were calculated to check the stability of each ACE2/2019-nCoV-Spike complex. Binding energy between the ACE2 protein and the 2019-nCov-Spike mutation system was calculated by using the MMPBSA module implemented in the AMBER software [28]. The binding interface region was defined as the contiguous protein surface region comprising all residues with at least one heavy atom within a distance < 4.5 Å from the associated protein [29]. Binding surface area calculation was conducted by counting the accessible surface area (ASA) using the typical “rolling ball” algorithm developed by Shrake and Rupley [30].

## Results

### Mutation sites on Omicron strain

**Figure. 1:**
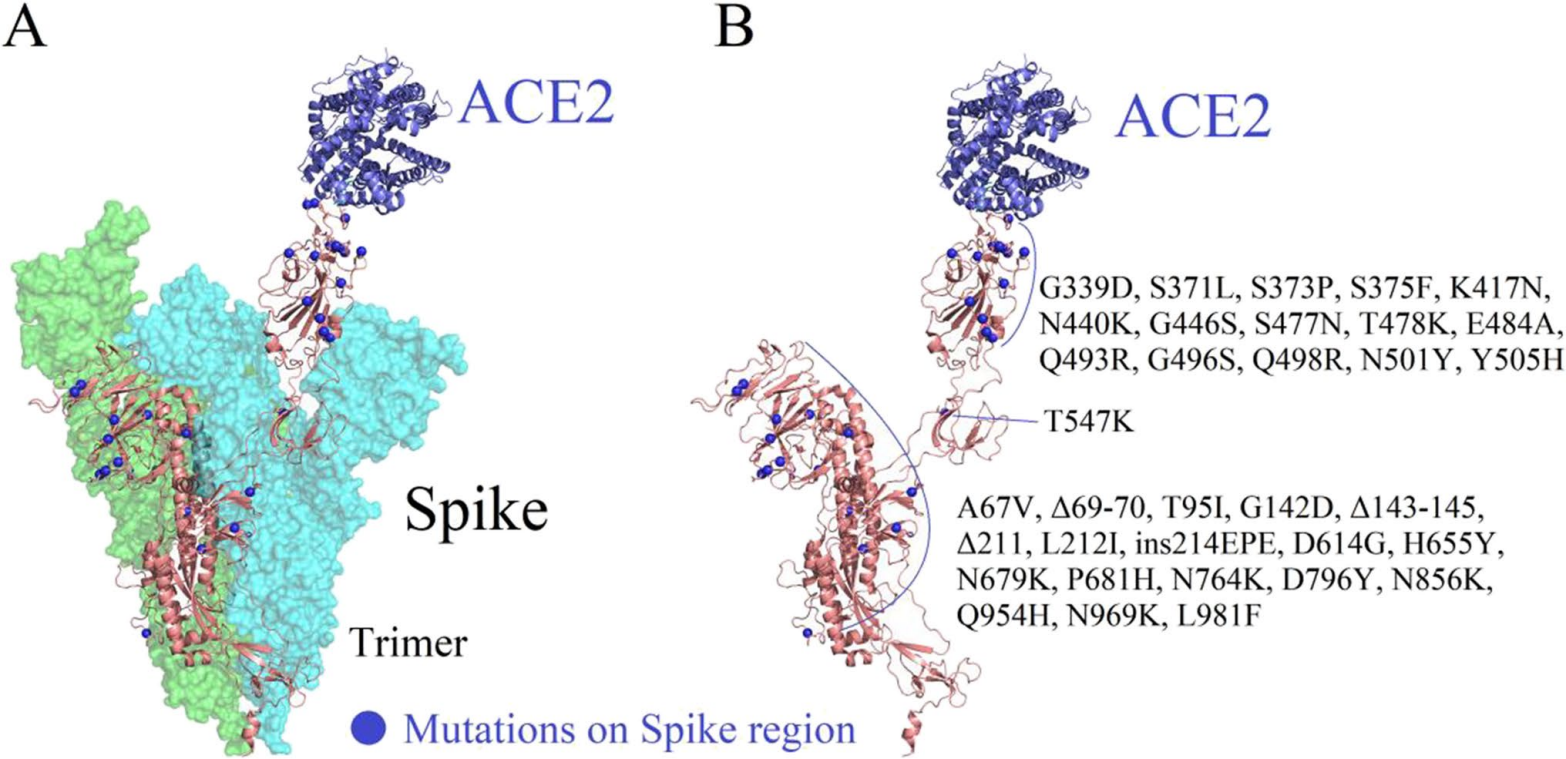
Distribution of 34 mutations on Omicron variant. **A** Overall structure of the Spike with human receptor ACE2. Schematic of 34 mutation sites on the Spike region is shown as sphere. **B** List of the selected 32 mutation sites on SARS-CoV chimeric receptor-binding domain complexed with its receptor human ACE2

The variant Omicron owns 60 mutations compared with the original Wuhan variant (Table 1 and Fig. 1). Among the mutations, 50 non-synonymous mutations, 8 synonymous, and 2 non-coding mutations were detected on the 2019-nCov virus, and 34 mutations were found to be distributed on the spike region. Interestingly, 3 small deletion mutations and 1 small insertion mutation and 15 single mutation are located in the 2019-nCov-Spike/ACE2 receptor-binding interface domain. It also carries some changes and deletions in other genomic regions. Interestingly, only one mutation was distributed in the envelope domain. In addition, this variant has 3 mutations on the membrane site. The ORF1b and nucleocapsid region also have 11 and 4 mutation sites, respectively [31].

**Table 1:** Distributions of mutation sites on Omicron variant

Region	Mutations
Spike	A67V, Δ69-70, T95I, G142D, Δ143-145, Δ211, L212I, ins214EPE, D614G, H655Y, N679K, P681H, N764K, D796Y, N856K, Q954H, N969K, L981F
ORF1ab	nsp3 (K38R, V1069I, Δ1265, L1266I, A1892T), nsp4 (T492I), nsp5 (P132H), nsp6 (Δ105-107, A189V), nsp12 (P323L), nsp14 (I42V)
Envelope	T9I
Membrane	D3G, Q19E, A63T
Nucleocapsid	P13L, Δ31-33, R203K, G204R

The Spike protein plays a critical role in identifying and binding the host cell surface receptors and mediating the fusion of viral envelope to the cell membrane. The spike protein is like a “key,” and the ACE2 receptor on the cell is like a “lock.” The key is locked for the virus to enter the cell. The main goal of developing the COVID-19 vaccine is also to prevent keys from opening locks to prevent the virus from infecting cells. Thus, learning mutation sites on Omicron spike proteins will supply an extremely important driving role for drug and vaccine development.

Currently, multiple crystal structures of the 2019-nCov-Spike/ACE2 complex have been resolved in the RCSB PDB database. In this paper, structure of the SARS-nCov-2 spike glycoprotein (closed state) with RCSB PDB ID: 6VXX [32] was selected, and excess elements including waters, ions, and peptides were deleted for structural studies. Spike protein belongs to the trimer with the top region on each monomer, which was capable of tightly binding to ACE2. All amino acids on each protein between the 2019-nCov-Spike protein and ACE2 within the 0.5-nm distance from each other were set as the binding interface.

As shown in Fig. 1A, amino acid mutation sites on the Spike region were uniformly distributed over the Spike region. Among them, the mutation sites which were adjacent to the ACE2-binding interface is more important for the stability of 2019-nCov/ACE2 complex due to the strong structural interference on the complex system. Here, we selected the 32 amino acid mutation sites on the Spike region for subsequent analyses. The detailed information of selected 32 mutation sites is listed in Fig. 1B.

### Structure constructions of 34 single-mutants and 1 multiple-mutant

The 3D structures of the wild-type 2019-nCov/ACE2 complex system was directly extracted from the crystal structure with PDB ID: 6VXX. Subsequently, single-point mutants based on the wild-type Spike protein were constructed by using the mutated wizard module on PyMOL software. A total of 34 single-point mutations were obtained.

### Binding energies of 35 mutants

Mutation of an amino acid on protein often causes the variation of biological function. We firstly performed primary molecular simulations for 34 single-point mutant and 1 multiple-mutant. We used the molecular dynamic software Amber 16 to conduct structural optimization for each 2019-nCov-Spike/ACE2 mutant complex. A force of 1500 kcal/mol was applied to all the heavy atoms on the Spike and ACE2 proteins during the MD simulations. The root mean square deviation (RMSD) of the heavy atoms of each multiple-mutant was examined until the MD simulations reached equilibrium. Binding free energies of the 2019-nCoV-Spike/ACE2 mutants were extracted for differential analysis. Each positional optimization time was at least 6 ns per system. Binding energy between the ACE2 protein and the 2019-nCov-Spike mutation system was calculated by using the MMPBSA module based on the 6-ns molecular dynamic simulation, as shown in Table 2.

**Table 2:**
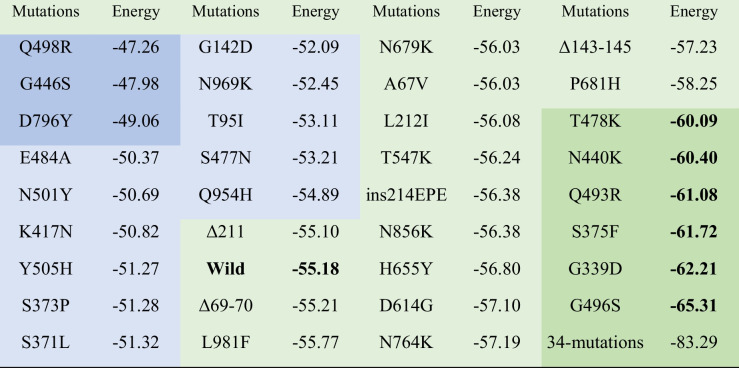
Binding energies (Kcal/mol) of 35 mutants 2019-nCov-Spike/ACE2 complex

Different colors presented the different binding energy intervals

### Six mutations triggered lower binding affinity of 2019-nCov-Spike to ACE2

The binding free energy of wild-type 2019-nCov-Spike/ACE2 protein complex was −55.18 kcal/mol. Among the 34 single-point mutant systems, a total of 16 single-point mutations with energy higher than −55.18 kcal/mol were T95I, G142D, Δ211, S371L, S373P, K417N, G446S, S477N, E484A, Q498R, N501Y, Y505H, D796Y, Q954H, and N969K. This result indicated that not all mutations that appeared in Spike region on 2019-nCov virus can lead to increased binding capacity with human ACE2 protein. For 12 mutations, the energy is almost unchanged compared with that of the wild-type complex, indicating that these mutation sites do not affect much of the binding patterns between the virus and human beings. In addition, 6 complexes (G339D, Q493R, G496S, S375F, N440K, and T478K) own lower than −60.00 kcal/mol, accounting for 17.14%, indicating that in both mutations G496S and G339D, the binding energies of simulated system were −65.31 kcal/mol and −62.21 kcal/mol, respectively. Crucially, the multiple-mutant system with 32 mutations on the Spike protein has a minimum energy of −83.29 kcal/mol, which is completely consistent with the expected results, because the Omicron variant has an extremely strong infectious capacity.

**Figure. 2:**
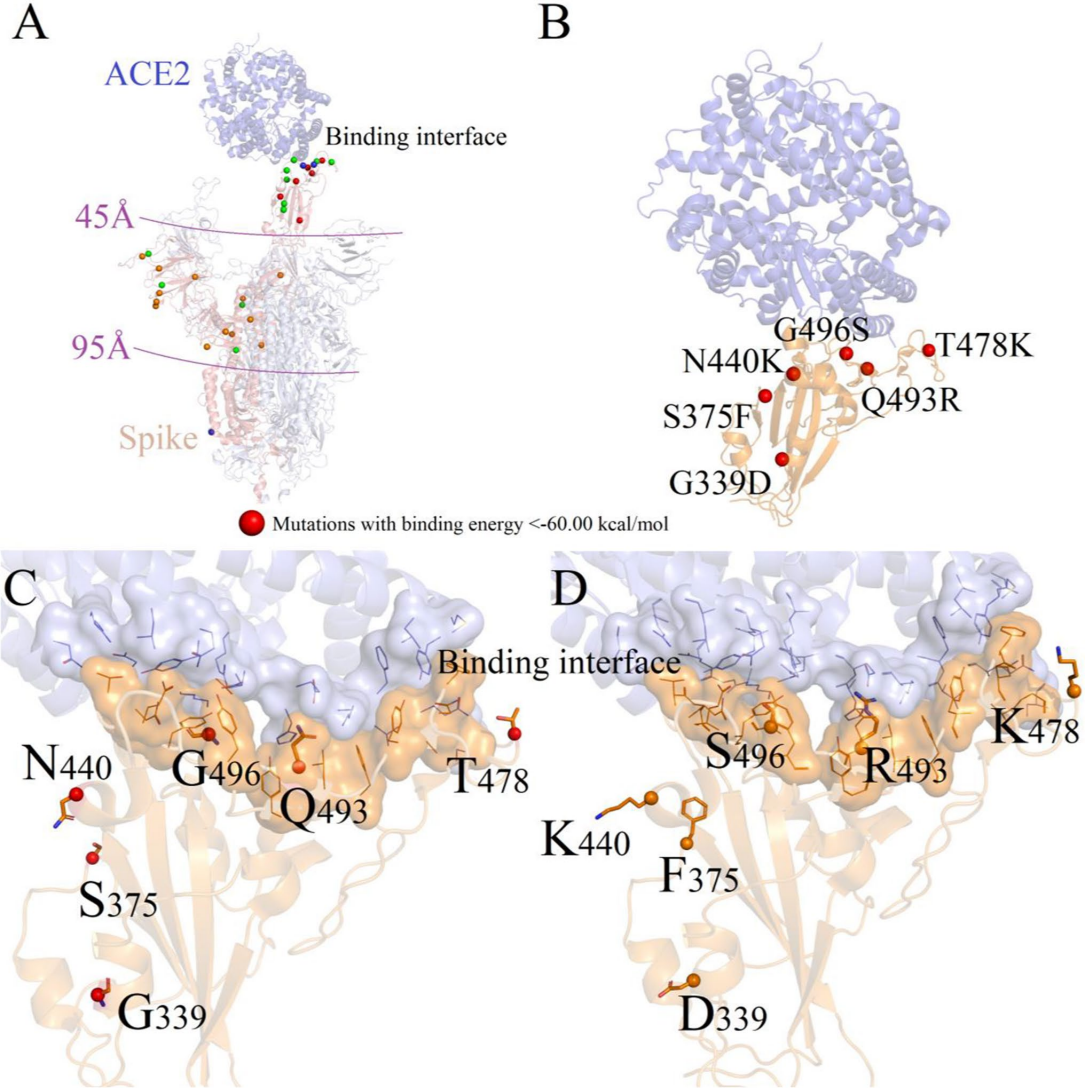
Positions of mutations with binding energies lower than −60.00 kcal/mol on Omicron variant

It can be seen from Fig. 2 that there being basic rules of the predicted energy distribution in the protein structure. Among them, the mutation sites possessing lower energies were almost close to the 2019-nCov-Spike/ACE2-binding interface. Mutation sites owning similar binding energies with that of wild-type Spike proteins are generally far away from the binding interface. More importantly, the 6 mutation sites with energy lower than −60.00 kcal/mol were all located on the Spike region, spread from G339 to Y505, among which the Q493R and G496S were directly involved in forming binding interface of 2019-nCov-Spike/ACE2 complex. All these results prompted us one deeper insight into the effects of mutations on the entire Spike protein structure and binding mode difference of 6 mutants (< −60.00 kcal/mol) and the multiple-mutant.

### 200-ns MD simulations for 6 single-mutant and 1 multiple-mutant

For the selected 6 single-point mutations (T478K, N440K, Q493R, S375F, G339D, and G496S) and 34-point mutation system, one 200-ns-long molecular dynamic simulations was conducted. Molecular dynamics were carried out by using the software Amber 16. Binding analysis and binding free energies for each system were calculated for protein-protein 2019-nCov-Spike/ACE2 complex after equilibrium phase based on the molecular dynamic trajectories.

The root mean variance (RMSD) represents the dispersion of centroid coordinates means, which can reflect the structural changes of the protein. During molecular dynamic process, the RMSD trend between initial structure and each time are monitored in real time, and the molecular dynamic simulation is stopped until the RMSD value reached stable.

**Figure. 3:**
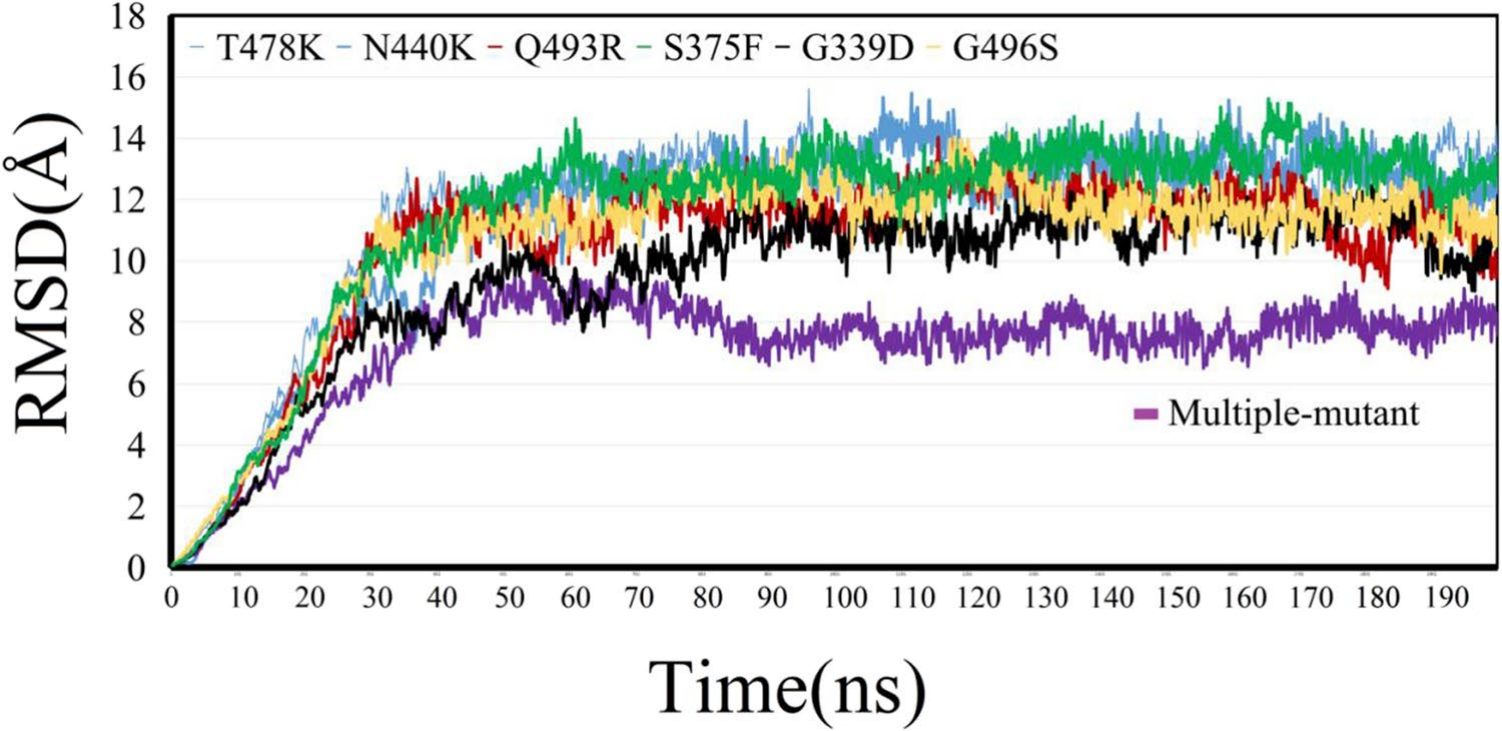
RMSD values for each single-mutant and multiple-mutant

As shown in Fig. 3, RMSD values for all the 6 single-point mutants exhibited large and drastic fluctuations, indicating the instability of virus to the human receptor ACE2. Each mutation can cause the apparently initial structural changes for 2019-nCov/Spike complex within the starting 1~30-ns time range, and the RMSD vales keep floating up and down, indicating the conformation changes when the virus binds to the human ACE2 protein. Within the 30~190-ns time range, RMSD values for 6 single-point mutations (T478K, N440K, Q493R, S375F, G339D, and G496S) maintained smoothly between 10 and 14 Å, indicating the relatively stable binding modes between Spike and the human ACE2 protein. Notably, compared with 6 single-point mutant systems, the multiple-mutant Omicron possessed relatively lower RMSD value. RMSD value for the multiple-mutant with 34-point mutations finally fluctuated from 6 to 8 Å. Relatively lower RMSD value, meaning the smaller structural alteration, also indicates the weaker conformation change of multiple-mutant system itself. All results imply the smaller swing of multiple-mutant system 2019-nCov-Spike/ACE2. It is interesting to note that the co-existing 34 mutations for Omicron strain does not trigger larger conformation change, but made itself relatively more stable to bind with human ACE2. Different binding free energies for mutants and wild-type Omicron 2019-nCov-Spike/ACE2 complex are shown in Table 3.

**Table 3:** Binding free energies (kcal/mol) between each mutant and ACE2 protein

System	∆E_VDWAALS_	∆Electronic	∆E_GB_	∆E_SURFACE_	∆E_GAS_	∆E_SOL_	∆E_TOTAL_
T478K	−95.29	−1308.66	1351.45	−13.88	−1403.94	1337.58	−66.37
N440K	−90.75	−1536.11	1573.50	−13.47	−1626.86	1560.03	−66.83
Q493R	−88.68	−1088.88	1123.61	−14.03	−1177.56	1109.58	−67.99
S375F	−89.21	−857.69	893.89	−13.40	−946.90	880.49	−66.41
G339D	−87.34	−1143.46	1177.78	−13.77	−1230.80	1164.01	−66.79
G496S	−95.48	−770.16	812.15	−13.60	−865.64	798.55	−67.09
34-mutations	−98.75	−962.51	990.70	−15.24	−1061.26	975.46	−85.80

*VDWAALS* = Van der Waals contribution from MM

*EEL* = electrostatic energy as calculated by the MM force field

*EGB* = the electrostatic contribution to the solvation free energy calculated by GB respectively

*ECAVITY* = nonpolar contribution to the solvation free energy calculated by an empirical model

DELTA G binding = final estimated binding free energy calculated from the terms above (kcal/mol)

### Difference analysis of binding modes

**Figure. 4:**
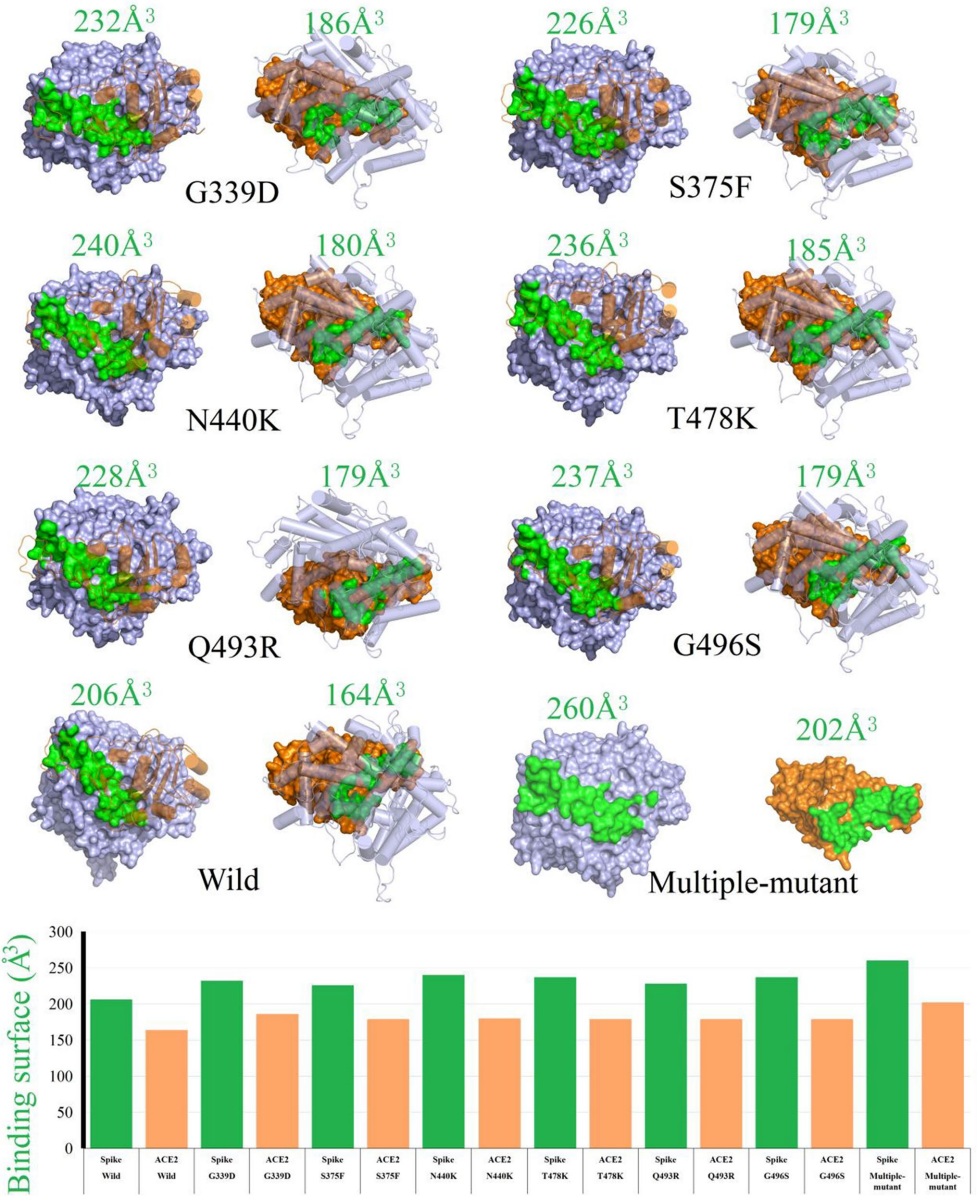
Identifying potential 2019-nCov-Spike/ACE2 binding interfaces. Binding interface are obtained on the surface of 2019-nCov-Spike (left) and ACE2 (right) for wild-type and each mutant complex. Binding surface of Spike and ACE2 are both colored as green. Binding surface area were counted

In order to elucidate the binding patterns of 2019-nCov-Spike to ACE2 protein in different mutants (6 single-point mutants and 34-mutations), we performed a comparative analysis of their structural difference especially the binding interfaces. Assessing the binding surface areas between these possible interfaces indicates that wild-type 2019-nCov-Spike wrap over ACE2 on the lowest level. The predicted interface here was consistent with the crystal structure of 2019-nCov-Spike/ACE2 (PDB ID 6VXX). According to the combined surface area values between 2019-nCov-Spike and human ACE2 protein, the wild-type mutant owns the minimum contacting surface area 206 Å^3^ (ACE2) and 164 Å^3^ (Spike), which corresponds with the lowest binding free energies of −55.18 kcal/mol, whereas for 6 single-point mutants T478K, N440K, Q493R, S375F, G339D, and G496S, the contacting surface areas were 179 (ACE2)–237 Å^3^ (Spike), 180 (ACE2)–240 Å^3^ (Spike), 179(ACE2)–228 Å^3^ (Spike), 179 (ACE2)–226 Å^3^ (Spike), 186 (ACE2)–232 Å^3^ (Spike), 179 (ACE2)–237 Å^3^ (Spike), and 202 (ACE2)–260 Å^3^ (Spike). Interestingly, the six single-point mutants possessed the lower binding energies within −66.37 to approximately −67.99 kcal/mol. Residues of the single-point mutant 2019-nCov-Spike protein forms more intensive binding interface than that of wild-type virus. What is more, among all mutants, the Spike protein of Omicron has the lowest binding capacity to the human ACE2 at −85.80 kcal/mol. As shown in Fig. 4, we extracted two surface area difference for both Spike and ACE2 binding patterns when compared to humans.

### Binding mode difference between human ACE2 and mutants

We next investigated how the mutation sites influence the regional structure of 2019-nCov-Spike to the human ACE2 protein and analyzed the important residues of 7 mutants which disturb the physicochemical properties of binding interface. The 6 single-point mutants were divided into two categories according to the distance from binding interface: (1) mutation sites not directly form the binding interface (G339D, S375F, and N440K); (2) mutation sites formed direct interactions with ACE2 (T478K, Q493R, and G496S).

**Figure. 5:**
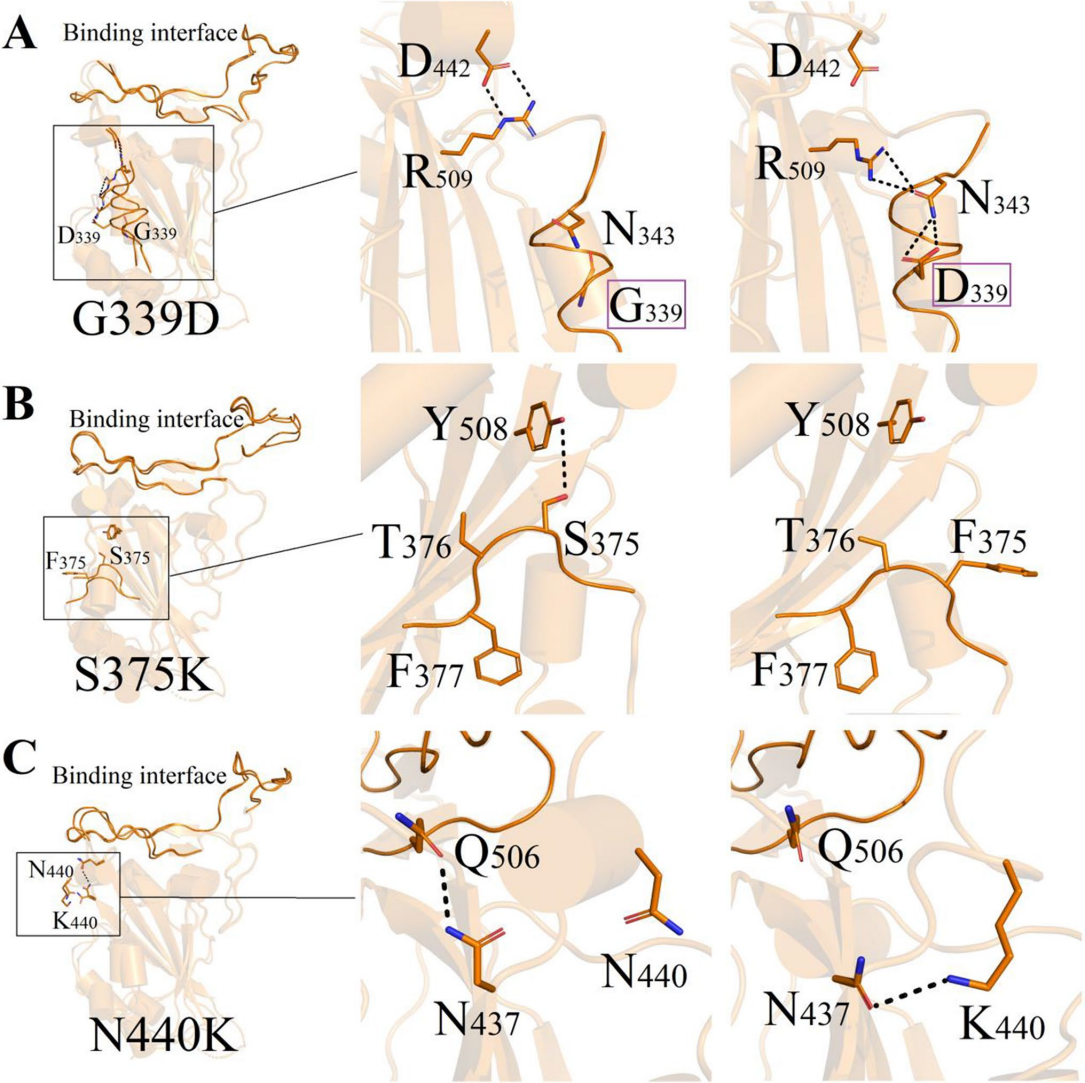
Binding difference of mutation sites between mutants (G339D, S375F, N440K) and wild-type coronavirus. Binding interface of 2019-nCov-Spike (left) and ACE2 (right) for wild-type and each mutant complex. Binding surface are shown as ribbon. Amino acids near mutation site are shown as sticks

**Figure. 6:**
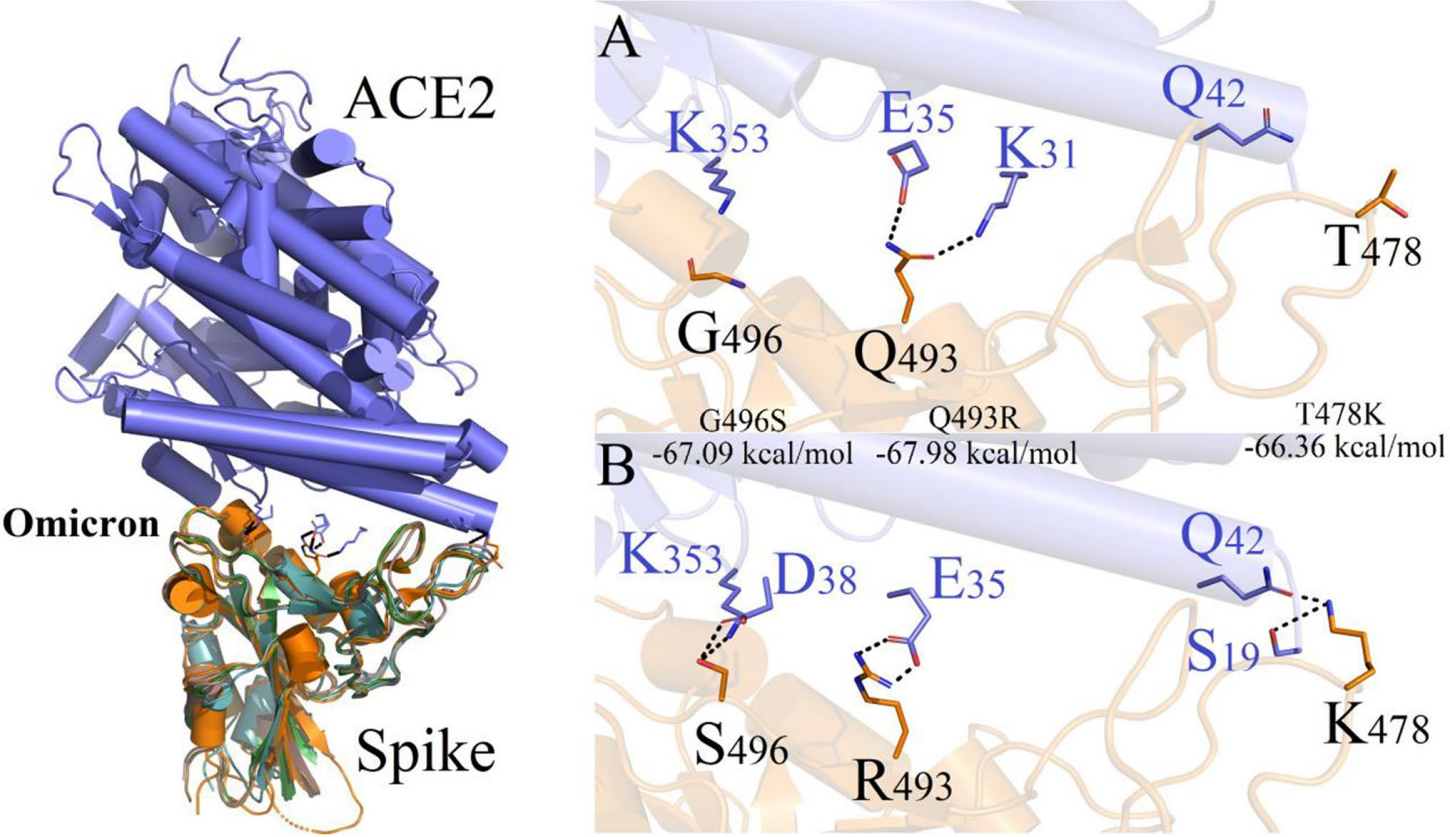
Binding difference of mutation sites between mutants (T478K, Q493R, G496S, and Omicron) and wild-type coronavirus. Binding interface of 2019-nCov-Spike (left) and ACE2 (right) for wild-type and each mutant complex. Amino acid near mutation site are shown as sticks

As shown in Fig. 5, mutation sites G339D, S375F, and N440K were all located far from the binding interface, and weak changes of mutation resulted in the conformation fluctuation near the mutation site. In mutant G339D, the system possessed relatively stronger binding capacity −66.79 kcal/mol than that of wild-type complex with −55.18 kcal/mol. The binding modes of ACE2 and 2019-nCoV-Spike are shown in Fig. 5A. Typical hydrogen bond interactions including N343-R509 and N343-D339 play significant roles in maintaining structural stability for mutant G339D. Figure 6 mapped the conformation differences at the same position S375/F375 between wild-type and mutant S375D. From Fig. 5B, we can see that one weak hydrogen bond interaction was formed between the hydroxyl groups of Y508 to S375 on Spike protein. Binding affinities for mutant S375F decrease obviously was −66.41 kcal/mol. This obvious conformation variation resulted the intensive contacting between mutant Spike and human ACE2 protein. A relatively higher energy variation (N440K, −66.83; wild-type, −55.18 kcal/mol) occurred due to the different binding modes for residues near mutation N440K. Binding mode data indicated that atoms on chemical group amin -NH3 from residue K440 make new hydrogen bonds with residue N437, as shown in Fig. 5C.

Similar results were also observed for mutants T478K, Q493R, and G496S. These 3 mutation sites were all located at the binding interface, and direct structural perturbations on the protein structure were observed in all 3 mutants. In wild-type coronavirus, only normal hydrogen bonds were formed between Q493 (Spike) and E35-K31 (ACE2). In the mutant G496S, amino acid S496 of Spike region formed good hydrogen bonds with the adjacent residues K353 and D38 on ACE2. However, mutated residue R493 from the mutant Q493R formed stronger salt-bridging interaction with opposite E35 amino acid. The same situations were also detected in mutant T478K, as new polar interactions were formed between the mutated K478 and Q42-S19 on human ACE2 protein. The key point was that residues K478, R493, and S496 on 34-points’ multiple-mutant Omicron strain simultaneously formed the same interactions as shown in single-point mutants T478K, Q493R, and G496S. Thus, the new mutated residues K478, R493, and S496 formed intensive polar interactions which significantly affect the structural stability of different mutants. Thus, we can infer that these three amino acids K478, R493, and S496 are crucial for high viral infection rates for mutant Omicron strain.

## Conclusions

The binding interface between the novel coronavirus Omicron 2019-nCov-Spike and human receptor ACE2 plays an important role in the viral infection. Various mutations, especially those mutations on 2019-nCoV-Spike region, can result in dramatic changes in viral transmission. Structural investigations of mutations on Omicron strain that affect the binding affinity of 2019-nCoV-S1/ACE2 complex can supply significant roles in drug and vaccine study and development. In this paper, we constructed protein-protein complex of 34 single-point mutants participating in forming Omicron strain, and then optimized the mutant systems by using molecular dynamic simulations. Comparing with the binding free energies of wild-type coronavirus Spike/ACE2 complex (−55.18 kcal/mol), 6 single-point mutants G339D, S375F, N440K, T478K, Q493R, and G496S possessed relatively lower binding free energy values. Binding mode difference analysis of residues K478, R493, and S496 on 34-points’ multiple-mutant Omicron strain played the key roles in forming intensive polar interactions that maintain structural stability of mutants. Thus, we can infer that these three amino acids K478, R493, and S496 are crucial for high viral infection rates for mutant Omicron strain.

## Data Availability

The data used to support the findings of this study are included within the article.

## References

[1] Xu Z, Shi L, Wang YJ, Zhang JY, Huang L (2020). Pathological findings of COVID-19 associated with acute respiratory distress syndrome. Lancet Respir Med.

[2] Yu P (2020). Age-related rhesus macaque models of COVID-19. Animal Model Exp Med.

[3] Lv Q (2020). Sensitivity of SARS-CoV-2 to different temperatures. Animal Model Exp Med.

[4] Ma Y (2021). SARS-CoV-2 infection aggravates chronic comorbidities of cardiovascular diseases and diabetes in mice. Animal Model Exp Med.

[5] Mahase E (2021). Covid-19: Pfizer’s paxlovid is 89% effective in patients at risk of serious illness, company reports. BMJ.

[6] Dyer O (2021). Covid-19: FDA expert panel recommends authorising molnupiravir but also voices concerns. BMJ.

[7] Andreadakis Z, Kumar A, Román RG, Tollefsen S, Saville M, Mayhew S (2020). The COVID-19 vaccine development landscape. Nat Rev Drug Discov.

[8] Le TT, Cramer JP, Chen R, Mayhew S (2020). Evolution of the COVID-19 vaccine development landscape. Nat Rev Drug Discov.

[9] Heath PT (2021). Safety and efficacy of NVX-CoV2373 Covid-19 vaccine. N Engl J Med.

[10] Chemaitelly H (2021). mRNA-1273 COVID-19 vaccine effectiveness against the B. 1.1.7 and B.1.351 variants and severe COVID-19 disease in Qatar. Nat Med.

[11] Arif TB (2022). The 501.V2 and B.1.1.7 variants of coronavirus disease 2019 (COVID-19): a new time-bomb in the making?. Infect Control Hosp Epidemiol.

[12] Angeletti S (2021). SARS-CoV-2 AY. 4.2 variant circulating in Italy: Genomic preliminary insight. J Med Virol.

[13] Espenhain L (2021). Epidemiological characterisation of the first 785 SARS-CoV-2 Omicron variant cases in Denmark, December 2021. Eurosurveillance.

[14] Ranjan, Prashant, Chandra Devi, and Parimal Das. “Bioinformatics analysis of SARS-CoV-2 RBD mutant variants and insights into antibody and ACE2 receptor binding.” bioRxiv (2021).

[15] Cameroni, Elisabetta, et al. “Broadly neutralizing antibodies overcome SARS-CoV-2 Omicron antigenic shift.” bioRxiv (2021). 10.1038/s41586-021-04386-2 PMC9531318 35016195

[16] COVID, CDC, and Response Team (2021). SARS-CoV-2 B. 1.1. 529 (Omicron) Variant—United States, December 1-8, 2021. Morb Mortal Wkly Rep.

[17] Dejnirattisai W (2021). Reduced neutralisation of SARS-CoV-2 omicron B. 1.1. 529 variant by post-immunisation serum. Lancet.

[18] He X (2021). SARS-Cov-2 Omicron variant: characteristics and prevention.

[19] Sahoo JP, Samal KC (2021). World on alert: WHO designated South African new COVID strain (Omicron/B. 1.1. 529) as a variant of concern. Biotica Research Today.

[20] Kannan SR (2022). Omicron SARS-CoV-2 variant: unique features and their impact on pre-existing antibodies. J Autoimmun.

[21] Ren S-Y (2022). Omicron variant (B.1.1.529) of SARS-CoV-2: mutation, infectivity, transmission, and vaccine resistance. World J Clin Cases.

[22] Chen J (2022). Omicron variant (B.1.1.529): infectivity, vaccine breakthrough, and antibody resistance. J Chem Inf Model.

[23] Kumar S (2022). Omicron and Delta variant of SARS-CoV-2: a comparative computational study of spike protein. J Med Virol.

[24] Šali A (1995). Evaluation of comparative protein modeling by MODELLER. Proteins: structure, function, and bioinformatics.

[25] Yuan S, Chan HS, Hu Z (2017). Using PyMOL as a platform for computational drug design. Wiley Interdisciplinary Reviews: Computational Molecular Science.

[26] Lindorff-Larsen K (2010). Improved side-chain torsion potentials for the Amber ff99SB protein force field. Proteins.

[27] Cazals F, Tetley R (2019). Characterizing molecular flexibility by combining least root mean square deviation measures. Proteins.

[28] Case DA (2005). The Amber biomolecular simulation programs. J Comput Chem.

[29] Vagenende V (2013). Quantifying the molecular origins of opposite solvent effects on protein-protein interactions. PLoS Comput Biol.

[30] Shrake A, Rupley JA (1973). Environment and exposure to solvent of protein atoms. Lysozyme and insulin. J Mol Biol.

[31] Khrustalev VV (2020). Translation-associated mutational U-pressure in the first ORF of SARS-CoV-2 and other coronaviruses. Front Microbiol.

[32] Rolta R (2020). Phytocompounds of Rheum emodi, Thymus serpyllum and Artemisia annua inhibit COVID-19 binding to ACE2 receptor: In silico approach. Current Pharmacology Reports.

